# Friend leukemia virus integration 1 activates the Rho GTPase pathway and is associated with metastasis in breast cancer

**DOI:** 10.18632/oncotarget.4350

**Published:** 2015-06-24

**Authors:** Wei Song, Wei Li, Lingyu Li, Shilin Zhang, Xu Yan, Xue Wen, Xiaoying Zhang, Huimin Tian, Ailing Li, Ji-Fan Hu, Jiuwei Cui

**Affiliations:** ^1^ Cancer Center, the First Hospital of Jilin University, Changchun, China; ^2^ Stanford University Medical School, VA Palo Alto Health Care System, Palo Alto, CA, USA; ^3^ Institute of Basic Medical Sciences, National Center of Biomedical Analysis, Beijing, China

**Keywords:** FLI1, breast cancer, metastasis, oncogene, RhoA pathway

## Abstract

Breast cancer is the most prevalent malignant disease in women worldwide. In patients with breast cancer, metastasis to distant sites directly determines the survival outcome. However, the molecular mechanism underlying metastasis in breast cancer remains to be defined. In this report, we found that Friend leukemia virus integration 1 (*FLI1*) proto-oncogene was differentially expressed between the aggressive MDA-MB231 and the non-aggressive MCF-7 breast cancer cells. Congruently, immunohistochemical staining of clinical samples revealed that *FLI1* was overexpressed in breast cancers as compared with the adjacent tissues. The abundance of FLI1 protein was strongly correlated with the advanced stage, poor differentiation, and lymph node metastasis in breast cancer patients. Knockdown of *FLI1* with small interfering RNAs significantly attenuated the potential of migration and invasion in highly metastatic human breast cancer cells. FLI1 oncoprotein activated the Rho GTPase pathway that is known to play a role in tumor metastasis. This study for the first time identifies *FLI1* as a clinically and functionally important target gene of metastasis, providing a rationale for developing *FLI1* inhibitors in the treatment of breast cancer.

## INTRODUCTION

Breast cancer is the most commonly diagnosed type of cancer and the second most fatal cancer of women in the world [1]. Over the last few decades, various targeted therapeutics have been introduced into the clinic, but there has been no corresponding improvement in patient survival, primarily because of the malignant behavior of breast cancer. Metastasis of breast cancer from primary location to distant sites is the main cause contributing to disease fatalities. However, factors controlling breast cancer cell migration remain to be elucidated. Therefore, it is urgent to identify key molecular regulators in metastasis to improve the prognosis assessment and treatment of breast cancer patients.

Friend leukemia virus integration 1 (*FLI1*) plays a critical role in normal development and hematopoiesis by functioning as both transcriptional activator and repressor [2–6]. The oncogenic role of *FLI1* was first confirmed in mice erytholeukemia [7]. Now, it is clear that about 85% of Ewing sarcoma cases are characterized by the presence of the *EWS/FLI1* fusion oncogene as a result of balanced chromosomal translocation t(11;22)(q24;q12). However, only recently has *FLI1* been reported to show aberrant activation in patients with solid tumors [8]. Despite these advances, little is known about the role of *FLI1* in metastasis, particularly in breast cancer.

In this communication, we examined the oncogenic activation of *FLI1* and its correlation with clinicopathological features in patients with breast cancer. Notably, FLI1 activated the Rho GTPase pathway that is known to be associated with metastasis in breast cancer.

## RESULTS

### Upregulation of FLI1 in metastatic breast cancer cells

To identify factors that are associated with metastasis, we selected an aggressive breast cancer cell line MDA-MB231 and a non-aggressive cell line MCF-7. We aimed to identify factors that were differentially expressed between these two cell lines.

**Figure 1:**
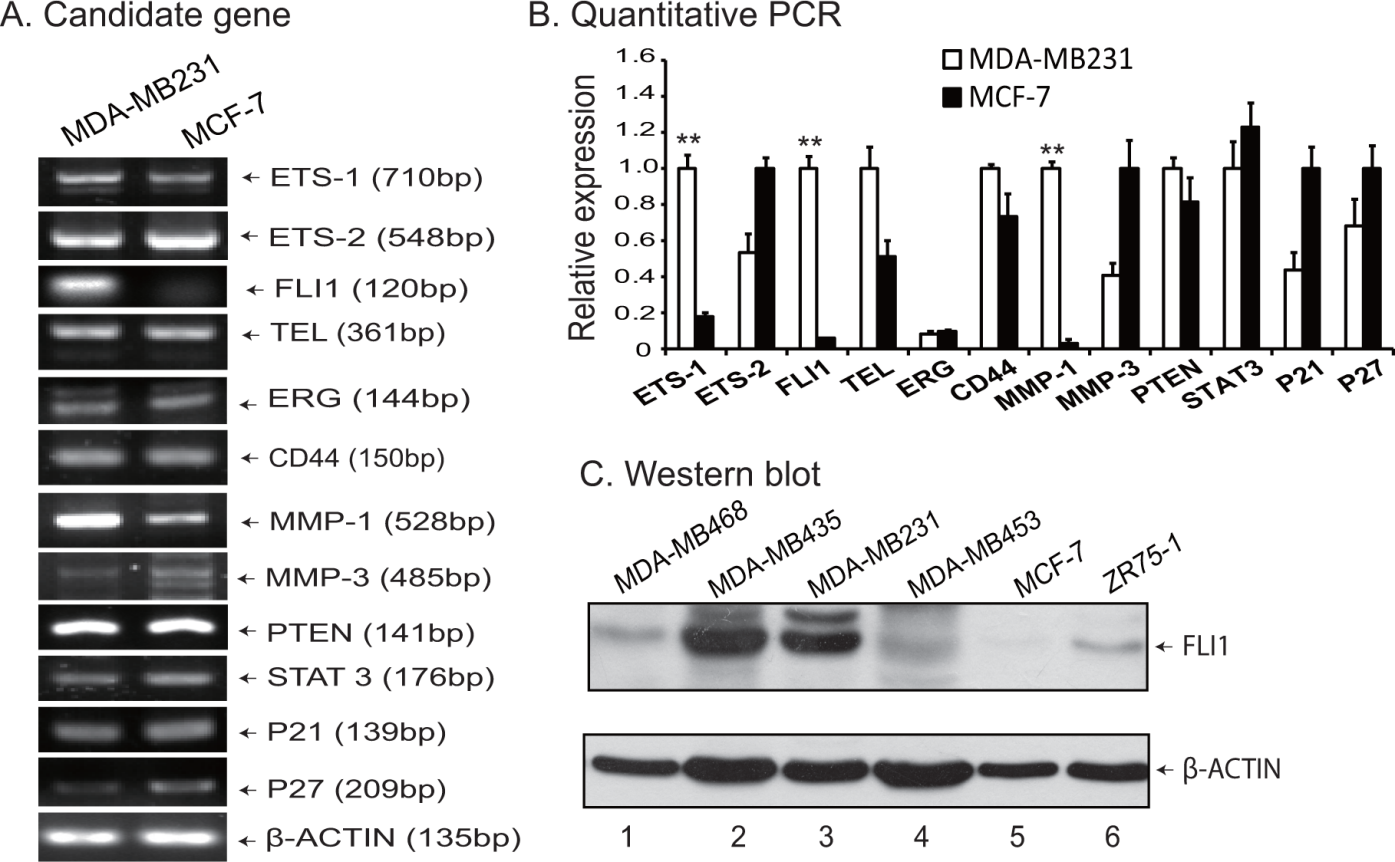
*FLI1* expression is associated with metastasis of breast cancer cells **A.** Representative RT-PCR data of genes analyzed. Differential expression of growth factors, oncogenes and target genes were compared between the aggressive MDA-MB231 and the non-aggressive MCF-7 cell lines. **B.** Confirmation of gene expression by quantitative PCR. Data shown are mean ± SEM from three independent experiments by normalization over the β-ACTIN control. **p < 0.01 as compared with MCF-7. **C.** Western blot of FLI1 in breast cancer cell lines.

Using RT-PCR, we analyzed the expression of growth factors, oncogenes and factors that may be related to tumor metastasis. Fig. 1A shows the typical data for part of candidate genes analyzed. Interestingly, we found that *FLI1* was one of the most notable genes that show distinct expression patterns. As a potential candidate target, *FLI1* was highly expressed in the aggressive MDA-MB231 cells, but was almost undetectable in the non-aggressive MCF-7 cells (Fig. 1A, lane 3). Quantitative PCR also confirmed the upregulation of *FLI1* in the aggressive MDA-MB231 cell line (Fig. 1B). In addition to *FLI1*, *ETS-1* and *MMP-1* were also significantly upregulated in the aggressive MDA-MB231 cells.

We then used Western blot to compare the expression of *FLI1* in five breast cancer cell lines that show varied metastatic abilities. As seen in Fig. 1C, *FLI1* was highly expressed in cell lines with high ability of metastasis, including MDA-MB231 and MDA-MB453 (lanes 2–3). Three breast cancer cell lines (MDA-MB468, MDA-MB453, and ZR75-1) exhibited weak expression of *FLI1*, while the non-aggressive MCF-7 was almost negative for *FLI1*.

### FLI1 activation correlates with breast cancer metastasis

**Figure 2:**
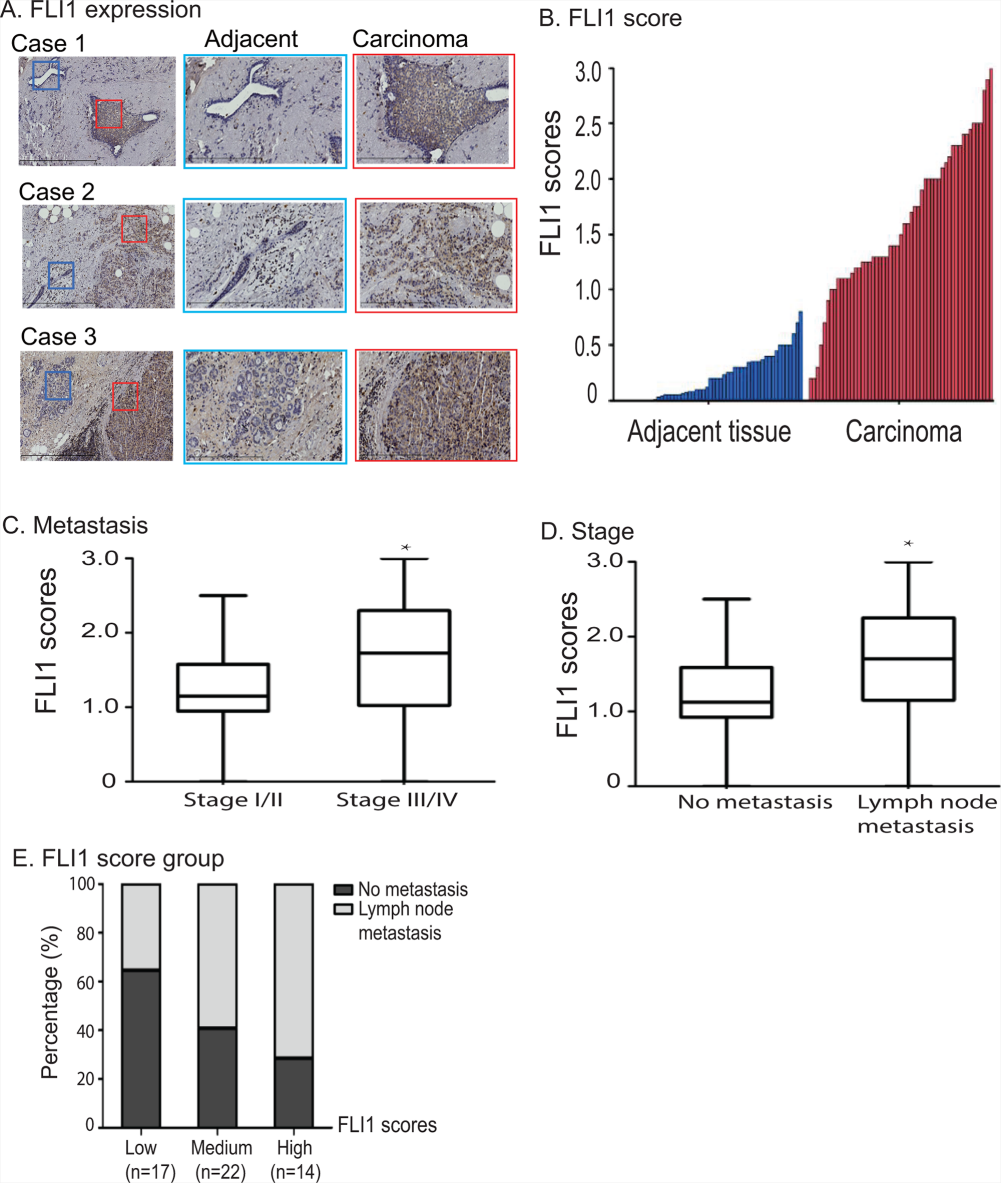
Overexpression of *FLI1* in breast cancer tissues **A.** Immunohistochemical staining of FLI1 in three representative breast cancer samples and their adjacent tissues. Blue square: adjacent normal tissue; red square: carcinoma. **B.** FLI1 is highly expressed in breast cancer tissues as compared with adjacent normal tissues. **C.** FLI1 expression scores in breast cancer patients in different clinical stages. **D.** High FLI1 expression in breast cancer patients with lymph node metastasis. **E.** Association between lymph node metastasis and FLI1 expression scores.

To determine the clinical significance of *FLI1*, we examined its expression pattern in tumor samples collected from breast cancer patients. Using immunohistochemically staining with FLI1 specific antibody, we found that FLI1 were significantly upregulated in breast tumor tissues as compared with adjacent normal tissues (Fig. 2A).

For comparison, we calculated the expression score for FLI1 and compared FLI1 score between breast cancer and adjacent tissues. There was a significantly higher FLI1 expression score in collected tumor tissues than that in their adjacent tissues (*P* < 0.01, Fig. 2B).

To better understand the role of *FLI1* in tumor prognosis, the association of FLI1 expression with stage and lymph node metastasis was also investigated. The FLI1 scores were higher in stage III/IV patients (1.7 ± 0.5) than that in stages I/II patients (1.2 ± 0.3)(Fig. 2C, *p* < 0.05). Similarly, FLI1 scores were also higher in patients with lymph node metastasis (1.7 ± 0.5) than that in patients without metastasis (1.1 ± 0.4)(*p* < 0.05)(Fig. 2D).

We divided FLI1 expression scores into three groups (low, medium, and high) and examined the percentage rate of breast tumor metastasis between groups. There was a clear positive association between the FLI1 expression score and the percentage rate of lymph node metastasis (Fig. 2E).

### Knockdown of FLI1 inhibits proliferation of breast cancer cells

To test whether *FLI1* is functionally important in breast cancer, we used two small interference RNAs (siFLI1 1# and siFLI1 2#) to knockdown *FLI1* in MDA-MB231 and MDA-MB453 cells. These two cell lines overexpressed *FLI1* and exhibited high metastatic potential.

**Figure 3:**
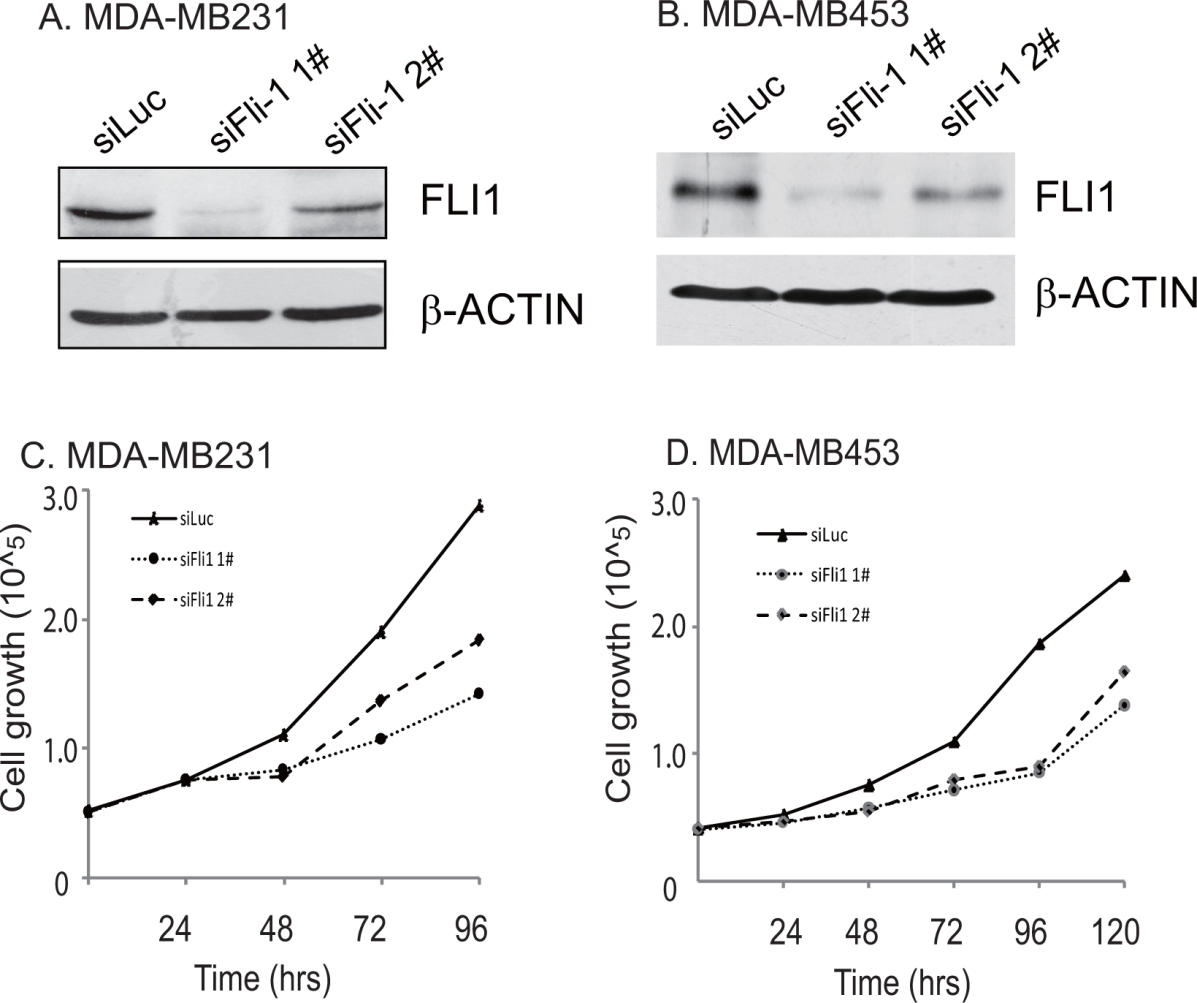
*FLI1* knockdown decreases cell proliferation in two aggressive breast cancer cells **A-B.** Knockdown of *FLI1* by two siRNAs (siFLI1 1#, siFli1 2#) in MDA-MB231 (A) and MDA-MB453 (B) *FLI1* expression was measured by Western blot. siLUC: control siRNA targeting the photinus pyralis luciferase gene. **C-D.** Inhibition of cell proliferation by *FLI1* siRNAs in MDA-MB231 (C) and MDA-MB453 (D) cancer cells.

Two siRNAs showed distinct activities in these two cell lines, with siFLI1 1# exhibiting a better inhibition potency than siFLI1 2# (Fig. 3A-3B). Correspondingly, we found a dose-dependent inhibition of cell proliferation with FLI1 knockdown in these two breast cancer cells (Fig. 3C-3D).

### Interference of FLI1 results in Go/G1 cell cycle arrest

**Figure 4:**
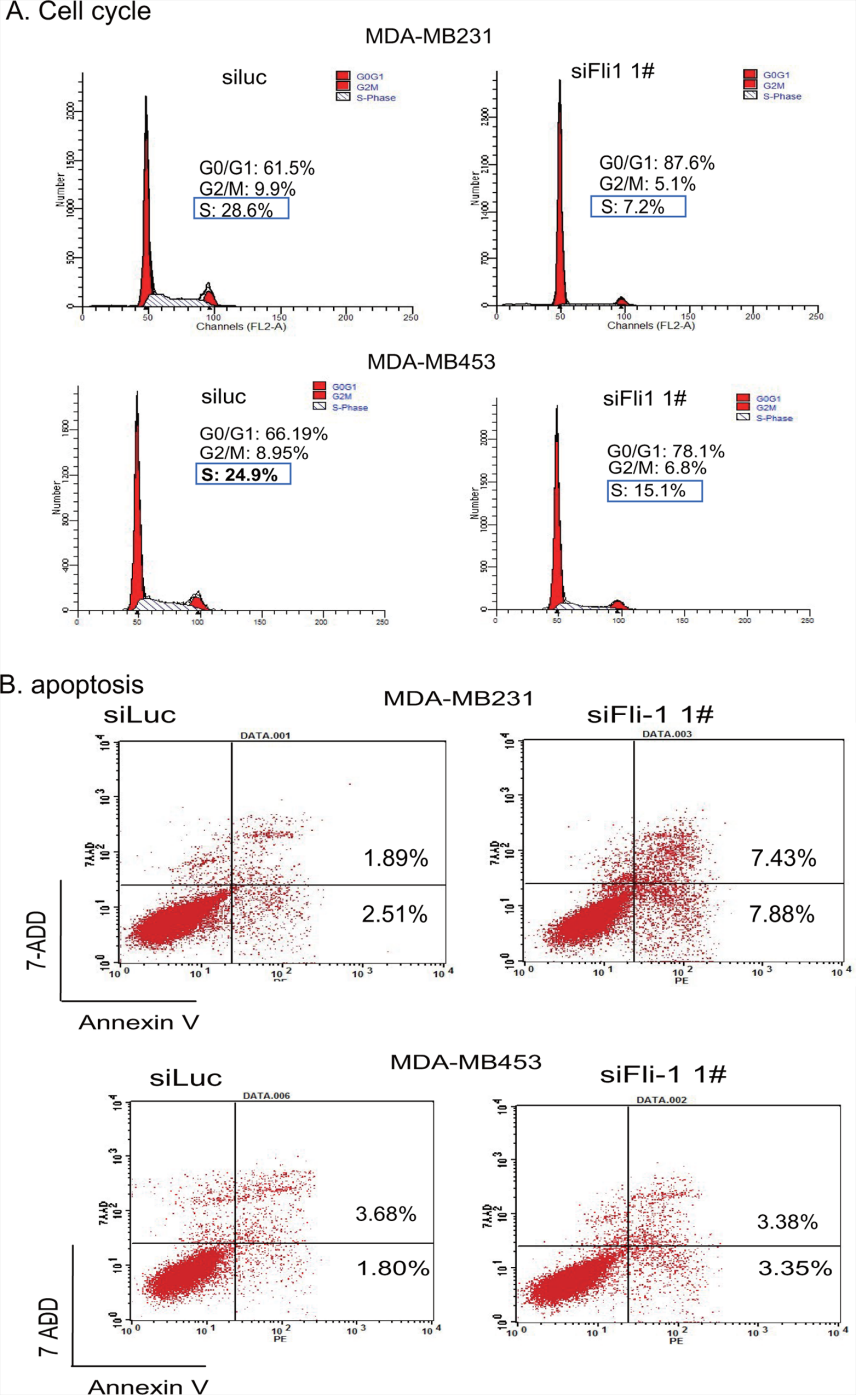
Analyses of cell cycle and apoptosis after interference of FLI1 expression **A.** Cell cycle in MDA-MB231 cells treated with the control siRNA (siluc) and *FLI1* siRNA (siFLI1 1#). **B.** Apoptosis in MDA-MB231 cells following the knockdown of *FLI1*.

To study the potential mechanism of *FLI1*, we analyzed cell cycle in the *FLI1*-knockdwon MDA-MB231 and MDA-MB453 cells. The cells in G0/G1 and S-phase were measured by flow cytometry (Fig. 4A). After *FLI1* knockdown, there was a dramatic decrease of cells in S-phase (28.6% vs 7.2% in MDA-MB231 cells and 24.9% vs 15.1% in MDA-MB453 cells). In parallel, there was an increment in the blockage of cells in the G0/G1 phase (61.5% vs 87.6% in MDA-MBA231 cells and 66.2% vs 78.1% in MDA-MB453 cells).

Cell apoptosis was also analyzed after the knockdown of *FLI1* in these two breast cancer cells (Fig. 4B). Using flow cytometry, we found that there was an increment in apoptosis cells in MDA-MB231 cells (top panel, 1.89% vs 7.43%), but this pattern was not notable in MDA-MB453 cells (bottom panel, 3.7% vs 3.4%).

### FLI1 is critical to maintain breast cancer metastasis

**Figure 5:**
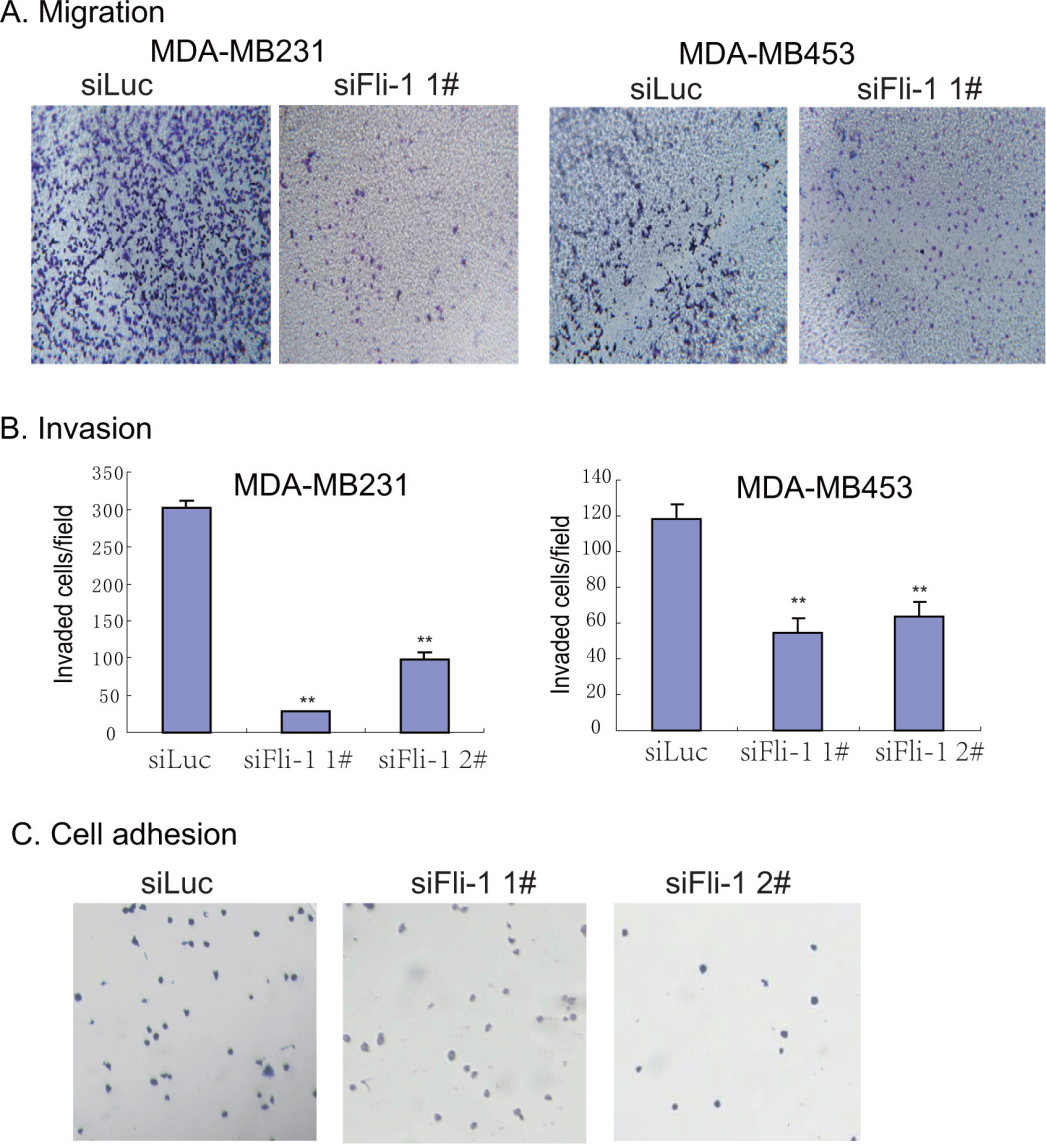
Knockdown of *FLI1* inhibits metastasis of breast cancer cells **A.** Reduced cell migration in MDA-MB231 and MDA-MB453 cells following the knockdown of *FLI1*. **B.** Cell invasion assay. Cells invaded through the collagen-coated membrane of the transwell were counted. All data shown are mean ± SEM from three independent experiments. *p < 0.05 as compared with control cells (siluc). **C.** Cell adhesion assay. Note the reduced ability of cell adhesion following the knockdown of *FLI1*.

The role of *FLI1* in breast cancer metastasis was evaluated by cell migration, invasion, and adhesion assays. To determine the role of *FLI1* in cell migration, we knocked down *FLI1* with siRNAs in two highly metastatic human breast cancer cell lines (MDA-MB231 and MDA-MB453). Using the transwell assay, we showed that knockdown of *FLI1* significantly decreased cell migration in both cell lines (Fig. 5A).

The role of *FLI1* in tumor cell invasion was examined by the collagen-coated transwell assay. In this assay, MDA-MB231 cells were allowed to invade through the collagen-coated trasnwell. As shown in Fig. 5B, we found that knocking-down of *FLI1* significantly reduced the invasion of both MDA-MB231 and MDA-MB453 cells in the 3D collagen invasion assay.

To further understand the role of *FLI1* in metastasis, we examined the capacity of cell adhesion in siFLI1 1#-treated cells. We found that MDA-MB231 cells treated with *FLI1* siRNA were much less adhesive than cells treated with the control siRNA (Fig. 5C). These data suggest that the upregulated *FLI1* may impair the cell adhesion phenotype.

### FLI1 promotes metastasis through the Rho GTPase pathway

**Figure 6:**
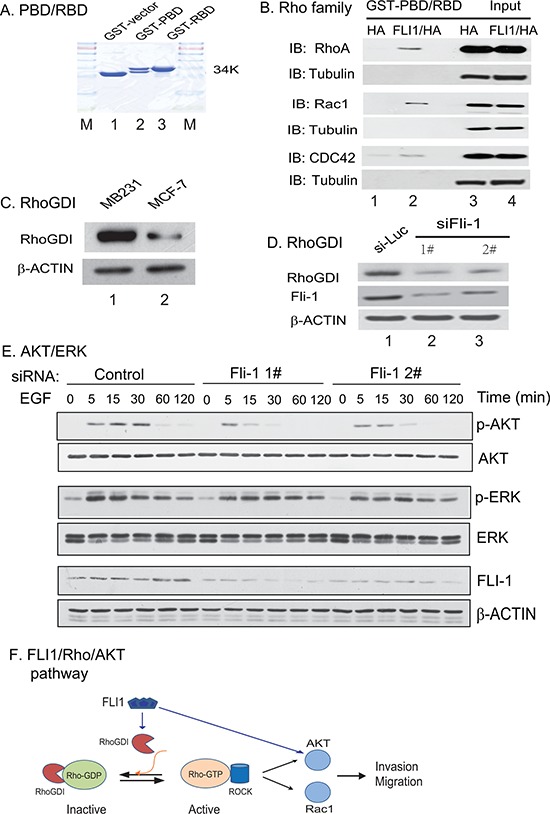
Activation of the Rho GTPase pathway **A.** GST-PBD and GST-RBD fusion proteins used for quantitate the active RhoA and Rac1. **B.** Detection of the active Rho GTPases by the GST-PBD and GST-RBD pull-down. PBD: the p21-binding domain (PBD) of the Rac1 effector p21-activated kinases (PAK); RBD: the RhoA binding domain (RBD) of the RhoA effector rhotekin. Note the activation of RhoA, Rac1, and CDC42 in FLI1/HA-expressing breast cancer cells (lane 2) as compared with that in the HA control (lane 1). **C.** Upregulation of RhoGDI in the aggressive MDA-MB231 breast cancer cells. D. Knockdown of *FLI1* reduces the Rho GDP-dissociation inhibitor (RhoGDI). **E.** Activation of the AKT/ERK cascade. Knockdown of *FLI1* decreases the activity of the AKT pathway. **F.** The proposed model of the FLI1/Rho/AKT pathway.

It is well established that Ras homologous (Rho) GTPases family proteins, like Rac1 and RhoA, play a key role in cell migration and tumor metastasis by regulating actin dynamics and cell-cell adhesion [9]. We thus investigated whether *FLI1* may play a role in metastasis by activating the Rho GTPase family genes. For this, we purified GST-PBD and GST-RBD proteins (Fig. 6A) and used them to detect the active Rac1 and RhoA [10, 11]. In this binding assay, the p21-binding domain (PBD) of the Rac1 effector p21-activated kinases was used to affinity precipitate the active Rac1, and the RhoA binding domain (RBD) of the RhoA effector rhotekin was used to affinity precipitate the active RhoA.

To investigate the role of *FLI1* in regulating Rho GTPase family protein function, we ectopically expressed *FLI1* in MDA-MB231 cells. The control cells were transfected with the HA control vector. GST-pull down assay and coimmunoprecipitation assay were used to detect the active Rac1 and RhoA. Using this binding assay, we found that the ectopically expressed *FLI1* was able to activate both Rac1 and RhoA GTPases, and enhanced the activity of CDC42 GTPase (Fig. 6B, lanes 1 vs 2).

Rho GDP-dissociation inhibitor (RhoGDI) is a critical regulator of Rho GTPase function [12]. Activation of Rho GTPase signaling pathways requires the regulated release of Rho GTPases from RhoGDI complexes, followed by their reuptake after membrane cycling. However, a clear picture of the role of RhoGDIs in breast cancer has not emerged. RhoGDIs can mediate both pro-tumorigenic and anti-tumorigenic signaling pathways [13]. We measured RhoGDI in our cell model using a Western antibody that recognizes both RhoGDI1 and RhoGDI2. We found that RhoGDI was overexpressed in the highly invasive MDA-231 cells as compared with the non-invasive MCF-7 cells (Fig. 6C). Similarly, FLI1 siRNA treatment dramatically downregulated RhoGDI (Fig. 6D, lanes 2–4), in parallel with the knockdown of FLI1 oncoprotein. These data suggest that *FLI1* may regulate the Rho GTPase pathway by controlling the activity of the RhoGDI inhibitor.

During cell migration, there is a close crosstalk between the Rho GTPase signal pathway and AKT/ERK cascades [14–16]. To examine this interaction in our model, we used EGF to stimulate cells that were treated with siFLI1 1# and siFLI1 #2. We found that the level of active phospho-AKT (p-AKT) was correlated with *FLI1* down-regulation (Fig. 6D). However, no such crosstalk was observed for the ERK cascade.

## DISCUSSION

Metastasis remains the primary cause of cancer-related death in patients with breast cancer. A comprehensive understanding of genetic determinants that promote metastatic dissemination is critical for the development and implementation of novel diagnostic and treatment strategies. In this study, we for the first time identify *FLI1* as a critical molecular factor associated with breast cancer metastasis.

*FLI1*, an Ets family member of transcription factors, was originally identified as a proto-oncogene for retroviral integration of Friend virus-induced erythroleukemias [17]. The EWS/FLI1 oncoprotein is formed by t(11;22)(q24;q12) rearrangement, in which *FLI1* becomes juxtaposed 3′ to the EWS gene [18]. EWS/FLI1 is detected in 95% of Ewing's sarcomas [19]. As a hallmark of Ewing's sarcoma, detection of this genetic alteration has provided a powerful tool in confirming the diagnosis of this childhood disease.

Oncogenic activation of *FLI1* leads to tumorigenesis, such as Ewing sarcoma and ovarian cancer. However, little is known about the role of *FLI1* in other solid tumors, particularly in breast cancer. In this study, we demonstrate that *FLI1* is overexpressed in cancer tissues as compared with the adjacent tissues. The *FLI1* expression score is closely correlated with clinicopathologic features, including advanced stages and lymph node metastasis. Consequently, knockdown of *FLI1* attenuates the metastatic potential in highly aggressive breast cancer cell lines. Our findings thus support a distinct paradigm for the involvement of *FLI1* in the progression of breast cancer.

It is well known that *FLI1* drives Ewing sarcoma through a mechanism of EWS-FLI1 fusion oncoprotein [20, 21]. By directly inducing or repressing gene enhancers, EWS-FLI1 establishes an oncogenic regulatory landscape in Ewing sarcoma governing both tumor survival and differentiation [22]. However, this EWS-FLI1 fusion mechanism is not active in breast cancer. Thus, the oncogenic role of *FLI1* in breast cancer metastasis, as observed in the present study, must be mediated through a distinct mechanism.

Rho GTPase comprise a major branch of the Ras superfamily of small GTPase, with Rac1, CdC42 and RhoA being best characterized as the important regulators of actin cytoskeleton organization, cell migration and cell progression. G proteins and their downstream signaling targets influence aberrant cell growth and survival, largely through the activation of AKT/mTOR, MAPKs, and Hippo signaling pathways [23]. Rho GTPases, including Rac1, CDC42 and RhoA, switch the cycle between inactive (GDP-bound) and active (GTP-bound) states [24]. During the complex process in tumor metastasis, tumor cell migration and invasion are two critical steps and responsible for the entry of tumor cells into blood vessels and lymph nodes, even the secondary tissues [25, 26]. In addition, cell adhesion, an important step in initiating tumor cell migration, is also tightly regulated by Rho GTPases [27]. The Rac signaling pathway is hyperactived in human breast cancer [28–30]. Constitutively activated Rac1 promotes focal adhesions in breast cancer cells. The activated Rac signal mediates breast cancer cell motility, invasion and breast cancer metastasis [30, 31].

We were thus interested in examining if *FLI1* affects the Rho GTPases pathway. Functional studies revealed that the forced expression of *FLI1* led to the activation of all three Rho GTPases: CDC42, Rac1 and Rho-A (Fig. 6B). Correspondingly, knockdown of *FLI1* decreased cell migration, invasion, and cell attachment (Fig. 5). Taken together, these data suggest that the activation of this signal pathway by *FLI1* may be associated with the metastatic potential of the breast cancer cells tested in this study.

The activities of Rho GTPases are regulated by RhoGDI [12]. The expression of RhoGDIs is altered in a variety of cancers. However, its specific role in various cancers remains controversial. RhoGDIs can mediate both pro-tumorigenic and anti-tumorigenic signaling pathways [13]. While early studies establish the role of RhoGDIs in maintaining Rho GTPase in inactive form [32], recent evidence has come to suggest that it may also act as a positive regulator in cancer progression. For example, RhoGDI was identified as an up-regulated protein in metastatic colorectal cancer [33], essential for cell proliferation, migration and distant metastasis. Similarly, Zhang et al reported that RhoGDI2 was highly expressed in tumor, but not in benign breast cell lines. Knockdown of RhoGDI2 in MDA-MB-231 cells resulted in decreased motility and invasion [34]. Ota et al also demonstrate that RhoGDIs positively regulate Rho GTPase activity by a distinct mechanism through direct interaction with GTPase activating protein (GAP) [35].

In this study, we found that RhoGDIs were upregulated in the aggressive MDA-MB-231 breast cancer cells as compared with that in the non-aggressive MCF-7 breast cancer cells (Fig. 6C). Knockdown of *FLI1* using shRNAs dramatically downregulated RhoGDIs (Fig. 6D), in parallel with the reduced migration and invasion of MDA-MB-231 breast cancer cells. Our data thus suggest that *FLI1* may regulate the Rho GTPase pathway in breast cancer, at least partially by controlling the activity of the RhoGDIs.

The Rho GTPases are also in a close crosstalk with the RAS-MAPK-ERK and PI3K-AKT-mTOR signaling pathways, which are the established factors controlling cell survival, differentiation, proliferation, metabolism, and motility [14–16, 36]. In normal cells, sustained activation of ERK1/ERK2 is necessary for G1- to S-phase progression and is associated with induction of positive regulators of the cell cycle [37]. Cellular processes regulated by AKT include cell proliferation and survival and tissue invasion. All these processes represent hallmarks of cancer [38]. Although both AKT and ERK pathways play an important role in malignant proliferation of breast cancer [39], our *FLI1*-transfection data only validate the role of p-AKT in MDA-MB231 and MDA-MB453 cells (Fig. 6D). p-AKT was significantly inhibited when *FLI1* was interfered, thus supporting the concept that *FLI1* may activate the AKT pathway in breast cancer cells.

In conclusion, this study for the first time demonstrates *FLI1* as a target gene that is associated with breast cancer metastasis (Fig. 6E). In this FLI1/Rho/AKT pathway, overexpression of *FLI1* in breast cancer activates the Rho GTPase family genes, either directly or by modulating the RhoGDI inhibitor. Through the pathway crosstalk, the AKT is activated to promote metastasis. Together, they induce a malignant phenotype that is associated with the metastasis of breast cancer. This study thus identifies *FLI1* as an attractive target for therapeutic intervention in breast cancer.

## MATERIALS AND METHODS

### Breast cancer samples

Formalin-fixed and paraffin-embedded tissues of breast cancer and control samples were obtained from the First Hospital of Jilin University between 2005 and June 2009. Primitive neuroectodermal tumor (PNET) was chosen as the positive control. Clinical data related to disease stage, histological grade and follow-up data were available for these patients. The pathological diagnosis was made in accordance with the histological classification of tumors developed by the World Health Organization. Tumor stage was defined according to American Joint Committee on Cancer/International Union Against Cancer classification system. Tumors were histologically graded according to the Elston and Ellis method. The study was approved by the Research Ethics Board of the First Hospital of Jilin University [40]. Informed consent was obtained from each breast cancer patient and normal subject. The clinical characteristics of the patients and the control subjects were shown in Table S1.

### Immunohistochemical staining

Tissue slides were deparaffinized with xylene and rehydrated through a gradual decline of alcohol (100–80%), and then incubated in 3% hydrogen peroxide for 15 minutes to block endogenous peroxidase activity. Antigen retrieval was carried out by immersing the slides in 10 mM sodium citrate buffer (pH 6.0) and maintained at a sub-boiling temperature for 15 minutes. The slides were rinsed in phosphate-buffered saline and incubated with 10% normal goat serum to block non-specific staining for 30 minutes at 37°C. Primary anti-FLI1 polyclonal antibodies (Neomarker) were diluted in 1:100, and incubated with the sections at 4°C overnight. After washing with PBS, the secondary antibodies (biotinylated goat anti-rabbit immunoglobulin) and streptavidin peroxidase complex reagent were applied. Subsequently, the visualization signal was processed according to the Polink-2 HRP DAB Detection kit. Finally, the slides were counterstained with hematoxylin for 15 min and dehydrated in ascending concentrations of alcohol (80–100%). After xylol treatment, slides were covered.

Two investigators were asked to evaluate each stained section independently without knowing any clinical information. The proportion of positive cells varied from 10 to 100%. Accordingly, the intensity of FLI1 staining was scored as 0 (negative), 1 (weak), 2 (moderate), and 3 (intense). The immunoreactivity score for each case was calculated as the percentage of positive cells per field multiplied by the intensity of staining.

### Knockdown of FLI1 by RNA interference

*FLI1*-specific siRNAs were purchased from Invitrogen (CA, USA), siFLI1 1#: 5′-GGGAAAGUUCAC UGUUGGCCUAUAA-3′ and siFLI1 #2: 5′-AGGAGU GGAUCAAUCAGCCAGUGAG-3′. The control siRNA (siluc) against photinus pyralis luciferase gene (Invitrogen, CA) was 5′-GGAUUUCGAGUCGUCUUAAUGU AUA-3′. RNAiMAX transfection reagent was used for transient transfection following manufacturer's protocol (Invitrogen, CA).

### Cell proliferation assay

Breast cancer cell lines (MDA-MB468, MDA-MB435, MDA-MB231, MDA-MB43, MCF-7, ZR75-1) were purchased from ATCC and were cultured in DMEM containing 1% penicillin and streptomycin, supplemented with 10% fetal bovine serum (FBS). For cell proliferation assay, cells were seeded in 96-well plates with density 10% per well. The number of cell proliferation was measured by Trypan-blue exclusion assay from day 1 to day 4.

### Cell migration and invasion assay

Cell migration and invasion assays were carried out using Transwell (Corning, MA) membrane filter inserted in 24-well tissue culture plates (6.5-mm diameter, 8-μm pore size) as previously described [41]. For migration assay, 4 × 10^4^ cells suspended in serum-free medium were seeded on the upper chamber of transwell filters. Serum-containing medium was added to the lower chamber and incubated for 16 h at 37°C. The non-migrating cells were removed by wiping the upper side of the filter, and the migrated cells on the bottom side of the filter were fixed with 4% formaldehyde, stained with crystal purple and counted under a microscopy.

A similar protocol was used for the invasion assay, except that cells were seeded in Transwell chambers coated with 0.5 μg/μl type I collagen (Invitrogen, CA, #A1048301) [41]. Each assay represents the average of three independent experiments.

### Western immunoblotting

Cells were lysed with immunoprecipitation assay buffer (1% Nonidet P-40, 50 mM Tris-HCl, pH 7.4, 150 mM NaCl, 1% sodium deoxycholate, 0.1% SDS, plus protease inhibitor cocktail and 1 mM phenylmethylsulfonyl fluoride). Proteins were separated by SDS-PAGE and analyzed by Western blotting. Antibodies to FLI1 and β-ACTIN were obtained from Santa Cruz Biotechnology (Santa Cruz Biotechnology, CA).

### RhoA GTPase pathway assays

The Rac1 activity assay was performed using the method as previously described [10]. The active Rac1 was quantitated by the affinity binding to a GST-PBD fusion protein, consisting of glutathione *S*-transferase (GST) and the p21-binding domain (PBD) of the Rac1 effector p21-activated kinases (PAK). Briefly, after washing with phosphate-buffered saline (PBS), cells were lysed immediately with PBD lysis buffer (50 mM Tris-HCl, pH 7.2, 100 mM NaCl, 5 mM MgCl2, 10% glycerol, 1% NP-40, 1 mM DTT and complete protease inhibitor Cocktail). Cell lysates were clarified by centrifugation at 16, 000 g at 4°C for 10 min. Equal volumes of lysates were incubated with GST-PBD fusion proteins on glutathione S-transferase beads to pull down active Rac1 proteins. After incubation at 4°C for 2 hours, the beads were washed three times with cold PBD lysis buffer. Rac1 protein was eluted with sample buffer and subjected to SDS-PAGE. Western blot was performed using anti-Rac1 antibody. Protein bands were visualized with an enhanced chemiluminescence reagent (Amersham Biosciences, PA).

The RhoA activity assay was performed as previously described [11]. The activated RhoA was determined by the binding to a GST-RBD fusion protein, consisting of glutathione *S*-transferase (GST)-RhoA binding domain (RBD) of the RhoA effector rhotekin. Briefly, the same protocol as in the above section was performed except using Rho-binding domain fused to GST (GST-RBD) and RBD lysis buffer (50 mM Tris-HCl, pH 7.2, 500 mM NaCl, 5 mM MgCl2, 1% Triton X-100, 1 mM DTT and Cocktail).

The activities of the AKT and ERK cascades were measured by Western blot as previously described [41].

### Statistical analysis

Comparisons between groups were analyzed by *t*-test. We assessed score comparisons between groups by one-way ANOVA test. *P* value of less than 0.01 was considered significant. Statistical calculations were performed using SPSS 13.0.

## SUPPLEMENTARY TABLE



## Abbreviations

BC: breast cancer
*FLI1*: friend leukemia virus integration 1
ROCK: rho-associated coiled coil-containing protein kinase
shRNA: short hairpin RNA
siRNA: small interfering RNA
