# Supplementary material for: Friend leukemia virus integration 1 activates the Rho GTPase pathway and is associated with metastasis in breast cancer
Source: Oncotarget. 2015 Jun 24;6(27):23764–75. doi: 10.18632/oncotarget.4350 (PMC4695150; doi:10.18632/oncotarget.4350)
Supplement: Supplementary file 1 [file oncotarget-06-23764-s001.pdf]

SUPPLEMENTARY TABLE

Supplementary Table S1. Clinicopathologic characteristics of breast cancer patients

Variable	Number	%
Age median (range)	48 (28–70)	
<48	26	49
≥48	27	51
TNM stage		
I	21	40
II	15	28
III	15	28
IV	2	4
Histological grade		
G1	3	6
G2	33	62
G3	17	32
Lymph nodal involvement		
Positive	31	58
Negative	22	42
ER expression		
Positive (>10%)	40	75
negative (≤10%)	13	25
PR expression		
positive (>10%)	34	64
negative (≤10%)	19	36
Her2 expression		
Positive	9	17
negative	44	83
